# Population pharmacokinetics of tigecycline in critically ill patients

**DOI:** 10.3389/fphar.2023.1083464

**Published:** 2023-03-13

**Authors:** Xiangru Luo, Shiyi Wang, Dong Li, Jun Wen, Na Sun, Guangjun Fan

**Affiliations:** Department of Pharmacy, The Second Affiliated Hospital of Dalian Medical University, Dalian, China

**Keywords:** critically ill patients, population pharmacokinetics, tigecycline, Monte Carlo simulation, dosage regimen

## Abstract

**Objective:** In critically ill patients, the change of pathophysiological status may affect the pharmacokinetic (PK) process of drugs. The purpose of this study was to develop a PK model for tigecycline in critically ill patients, identify the factors influencing the PK and optimiz dosing regimens.

**Method:** The concentration of tigecycline was measured LC-MS/MS. We established population PK model with the non-linear mixed effect model and optimized the dosing regimens by Monte Carlo simulation.

**Result:** A total of 143 blood samples from 54 patients were adequately described by a one-compartment linear model with first-order elimination. In the covariate screening analysis, the APACHEII score and age as significant covariates. The population-typical values of CL and Vd in the final model were 11.30 ± 3.54 L/h and 105.00 ± 4.47 L, respectively. The PTA value of the standard dose regimen (100 mg loading dose followed by a 50 mg maintenance dose at q12 h) was 40.96% with an MIC of 2 mg/L in patients with HAP, the ideal effect can be achieved by increasing the dosage. No dose adjustment was needed for *Klebsiella pneumoniae* for AUC0–24/MIC targets of 4.5 and 6.96, and the three dose regimens almost all reached 90%. A target AUC0–24/MIC of ≥17.9 reached 100% in patients with cSSSI in the three tigecycline dose regimens, considering MIC ≤ 0.25 mg/L.

**Conclusion:** The final model indicated that APACHEII score and age could affect the Cl and Vd of tigecycline, respectively. The standard dose regimen of tigecycline was often not able to obtain satisfactory therapeutic effects for critically ill patients. For patients with HAP and cIAI caused by one of three pathogens, the efficacy rate can be improved by increasing the dose, but for cSSSI infections caused by *Acinetobacter baumannii* and *K. pneumoniae*, it is recommended to change the drug or use a combination of drugs.

## 1 Introduction

Tigecycline is a new type of glycylcycline antibiotic that inhibits bacterial protein synthesis by binding to the 30S subunit of ribosome to prevent aminoacylated tRNA molecules from entering the A-site of the ribosome. It has a wide antibacterial spectrum and has shown good antibacterial activity against common pathogens and drug-resistant bacteria, including multidrug-resistant and extensively drug-resistant pathogens. It is therefore also considered as an anti-infective drug for critically ill patients in the intensive care unit (ICU), although pharmacokinetics (PK) data for this group are scarce (Kim et al., 2016; Xie et al., 2017; Papadimitriou-Olivgeris et al., 2018). The Food and Drug Administration (FDA) approved it for the treatment of complicated skin and soft tissue infection (cSSSI), complex abdominal cavity infection (cIAI) and community acquired bacterial pneumonia (Yaghoubi et al., 2022). It is also widely used to treat hospital-acquired pneumonia (HAP), urinary tract infection, blood flow infection and other diseases (De Pascale et al., 2014; Wang et al., 2017; Ben Mabrouk et al., 2021). The FDA issued a black-box warning that tigecycline can increase the mortality risk, but the reason for the high mortality rate has not yet been determined (Dixit et al., 2014; Jean et al., 2016).

Clinical studies have characterized the PK properties of tigecycline by its high rate of binding with plasma proteins (71%–89%) and atypical non-linear protein binding. Tigecycline has a large distribution volume in its steady state of about 500–700 L (7–9 L/kg), suggesting that its distribution volume in the tissue exceeds that in plasma. Tigecycline is also not widely metabolized in the body (Muralidharan et al., 2005; Pai, 2014). The main excretory pathway is bile secretion of the tigecycline prototype and its metabolites, and the secondary pathway is glycosylation and renal excretion of the tigecycline prototype (Yamashita et al., 2014).

In critically ill patients, pathophysiological changes may affect drug PK, thus affecting the required dose (Blot et al., 2014; Borsuk-De Moor et al., 2018; Broeker et al., 2018). PK changes in patients with severe illness include changes in the clearance (CL) rate caused by increased cardiac output or organ failure, and changes in distribution volume (Vd) caused by increased vascular permeability or changes in protein binding (Bastida et al., 2022). The status of patients will change according to disease development. The use of a standard dose of antibacterial drugs in ICU patients may lead to insufficient concentrations of the target drugs, which will lead to insufficient antibacterial activity and negatively impact outcomes (Montravers et al., 2014; Ibrahim et al., 2018). Therefore, it is necessary to quantify the relationship between patient covariates and pharmacokinetic parameters so as to achieve individualized administration. It is therefore necessary to quantify the relationship between patient covariates and PK parameters so as to achieve individualized administration. Early pharmacodynamics (PD) studies found that the ratio of the area under the 24 h curve to the pathogen MIC (AUC_0–24_/MIC) was the optimal pharmacokinetics/pharmacodynamics (PK/PD) target for tigecycline, but the specific target differed depending on the type of infection (Koomanachai et al., 2009). Previous studies found that the AUC_0–24_/MIC breakpoints were 17.9 for cSSSI, 6.96 for cIAI and 4.5 for HAP (Van Wart et al., 2006; Passarell et al., 2008; Kuti et al., 2019). However, the dose required to achieve these goals in ICU patients has not been investigated in detail.

The purpose of this study was to collect the blood concentration data and clinical information of patients treated in the ICU of a tertiary hospital, establish a PK model for tigecycline in ICU patients, determine the relationship between patient characteristics and PK parameters and evaluate the treatment effect of three infections under different drug regimens, so as to propose dose adjustment and provide a basis for promoting rational clinical drug use.

## 2 Materials and methods

### 2.1 Research population

This study retrospectively collected the information of 54 patients with severe illness from who used tigecycline to fight infection at the Second Affiliated Hospital of Dalian Medical University. The sample collection time was from December 2017 to July 2018. All patients were treated with a standard regimen of tigecycline (100 mg loading dose, 50 mg maintenance dose, q12 h). Inclusion criteria of patients were as follows: (1) received tigecycline intravenous infusion for more than 3 days, (2) male or female and aged ≥18 years, (3) clinically confirmed or suspected infection caused by G^+^ and G^−^ bacteria and (4) the blood concentration of tigecycline had been monitored. The exclusion criteria were (1) tigecycline was used for prevention, (2) the plasma concentration of tigecycline was not monitored during the treatment, (3) death occurred within 24 h of using tigecycline, (4) pregnancy, (5) known allergy to tigecycline or (6) incomplete clinical data. This research was approved by the Ethics Committee of the Second Affiliated Hospital of Dalian Medical University (2019 no. 049).

### 2.2 Clinical data

The baseline characteristics of patients were obtained from the electronic clinical records of the hospital. The information collected included 1). basic characteristics of patients (e.g., sex, age, weight); 2). laboratory test indicators (e.g., white blood cells, albumin, alanine aminotransferase, aspartate transaminase, blood creatinine); 3). certain types of infection and associated diseases; and 4). adverse reactions possibly caused by drugs such as hepatotoxicity, nephrotoxicity and anaphylaxis.

### 2.3 Blood sample collection and concentration determination

When the blood concentration of tigecycline reached a steady state after application, blood samples were collected by collecting residual blood, which would not cause secondary injury to patients. The sampling points were before administration and 1, 2, and 4 h after administration. The blood samples were centrifuged at 1.295 × 10^5^ g (TGL-16M desktop high-speed centrifuge, Shanghai Anting Scientific Instrument Factory) for 8 min to separate the supernatant and then stored in a refrigerator at −80°C for testing. The plasma concentration of tigecycline was determined using the liquid chromatography–mass spectrometry method established by our research group. The method was stable and reliable according to validation of its specificity, precision, accuracy, recovery and stability. Tigecycline had a strongly linear relationship over the concentration range of 1–2000 ng/mL (*R* ^2^ = 0.9907), the minimum detection limit was 10 ng/mL, and the relative standard deviations of intra- and interday precisions were 4.15% and 2.74%, respectively.

### 2.4 PK modelling

The plasma concentration data of tigecycline were fitted using a non-linear mixed-effects model (NONMEM 7.3.0, ICON Development Solutions, Hanover, Maryland, United States), and the PK parameters were estimated. The first-order interaction condition estimation (FOCE-I) method was used to estimate parameters, and the fixed effect parameters were CL and Vd. The modelling process included 1). preparing data files, 2). establishing a basic model, 3). establishing a statistical model, 4). establishing a covariate model, and 5). evaluating and verifying the models. Demographic data and biological indicators (including age, weight, AST, and albumin) were included as covariates in the model for testing. The covariates with objective function value (OFV) values that decreased by more than 3.84 (*p* < 0.05, df = 1) were retained by using the forward inclusion method. All of the covariates that had been retained were then eliminated one by one using the reverse elimination method. The covariates with OFV values that changed by more than 6.64 (*p* < 0.01, df = 1) were retained. Finally, the full regression model is obtained.

### 2.5 Model evaluation

The accuracy and applicability of the final model were evaluated by determining goodness of fit (GOF), which mostly focused on the GOF of observation concentration-population prediction concentration (PRED) and observation concentration individual prediction concentration (IPRE) scatter plots, PRED-conditional weight residuals (CWRES) and time-vs.-CWRES scatter plots. Ideally, these values should be evenly distributed on the Y = *X*-axis, with closeness to the axis indicating a more-accurate model fit. If the model fits well, CWRES should be symmetrically distributed on both sides of the Y = 0 reference line, most of which were within −2 and +2 and did not show obvious change trends with time. Bootstrapping, visual predictive check (VPC) and normal predictive distribution errors (NPDEs) could also be used to further verify the accuracy and predictability of the model.

### 2.6 Dose simulation

We used Monte Carlo simulation to assess the attainment of three AUC_0–24_/MIC targets that were derived from different types of infections (≥17.9 for cSSSI, ≥6.96 for cIAI and ≥4.5 for HAP) according to MIC distributions from european committee on antimicrobial susceptibility testing (EUCAST). Three different dose regimens were simulated: 1). 100 mg loading dose followed by a 50 mg maintenance dose at q12h, 2). 100 mg loading dose followed by a 75 mg maintenance dose at q12 h and 3). 200 mg loading dose followed by a 100 mg maintenance dose at q12 h. The simulation was performed 5,000 times with 95% confidence intervals (CIs) calculated to obtain the probability of target attainment (PTA) value, and the cumulative fraction of response (CFR) was then calculated. The treatment was considered effective if PTA was ≥90%. AUC_0–24_ was calculated as the ratio of the total tigecycline dose within 24 h to the total CL of the individual.

### 2.7 Data analysis

SPSS (version 22.0) software was used for descriptive statistical analyses, and the data were expressed as means and standard deviations or medians and quartiles. NONMEM (version 7.3.0) software was used for PPK analysis, and R (version 3.4.0) software and GraphPad Prism (version 8.0) were used for mapping.

## 3 Result

### 3.1 Demographic data

The study included 54 infected patients, and 143 blood drug concentrations were measured. The median age of patients was 72.0 years (57.5–80.3 years), and they comprised 30 males and 24 females. The median observed concentration was 444.0 ng/mL (222.3–716.6 ng/mL). Table 1 lists the basic clinical data of the patients.

**TABLE 1:** Demographic characteristics and clinical data of ICU patients.

Demographic characteristics	Number of patients or median value (IQR)
Male/female	30 vs. 24
Age (years)	72.0 (57.5–80.3)
Weight (kg)	68.0 (58.3–70.0)
ALT (U/L)	29.09 (18.70–63.73)
AST (U/L)	33.37 (21.80–77.15)
ALP(U/L)	89.30 (62.60–136.1)
TB (mmol/L)	16.22 (9.88–24.08)
Scr(μmol/L)	77.49 (53.98–121.7)
BUN(mmol/L)	9.30 (5.99–13.18)
ALB (g/L)	27.91 (25.58–32.50)
APACHE II	22.50 (16.50–27.00)
Na^+^(mmol/L)	137.0 (133.9–141.6)
Antifungal therapy	28
Number of modeling	54
Sample size	143
Observed concentration (ng/mL)	444.0 (222.3–716.6)

ALT, alanine aminotransferase; AST, aspartate aminotransferase; ALP, alkaline phosphatase; TB, total bilirubin; APACHE II, Acute physiology and chronic health evaluation II score; ALB, albumin.

### 3.2 Population pharmacokinetic analysis

A one-compartment linear model fully described the concentration–time process of tigecycline. The index model was the best for both the interindividual variation and residual variation models. In the covariate screening analysis, the Acute Physiology and Chronic Health Evaluation II score (APACHEII) was found to have a significant impact on the CL of tigecycline, and age had a significant impact on the Vd. The population-typical values of CL and Vd in the final model were 11.30 L/h and 105.00 L, respectively. The model was described as follows:

$$CL\left(\frac{L}{h}\right)=\left(11.30-0.14\times APACHE\;II\;score\right)\times e^{0.065}$$ (1)

$$V\left(L\right)={\left[105.00\times \left(1-0.0059\times AGE\right)\right]\times e}^{0.160}$$ (2)

**FIGURE 1:**
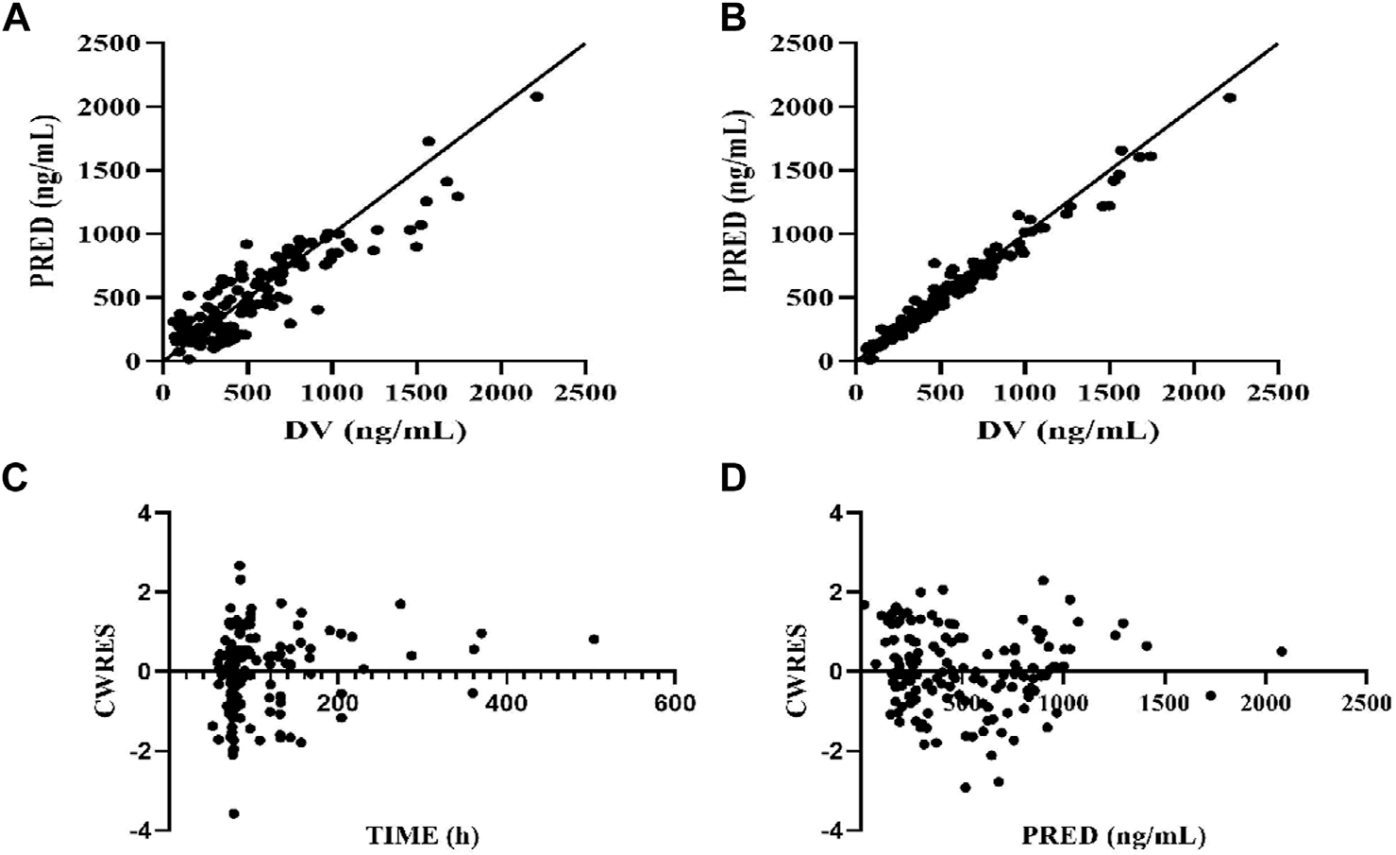
Diagnostic plot of final population pharmacokinetic model. **(A)** Scatter plot of observed values-population predicted values, **(B)** Scatter plot of observed values-individual predicted values, **(C)** Scatter plot of conditional weighted residuals—time since initial dose, **(D)** Scatter plot of conditional weighted residuals-predicted values of the population. The solid line is reference line.

**FIGURE 2:**
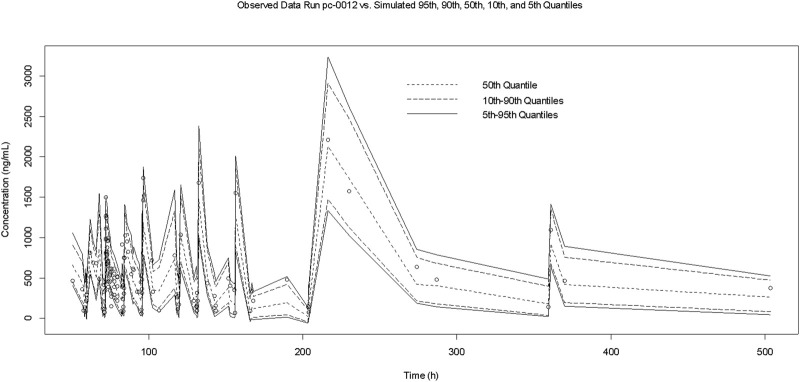
VPC graph of the final popPK mode.

**FIGURE 3:**
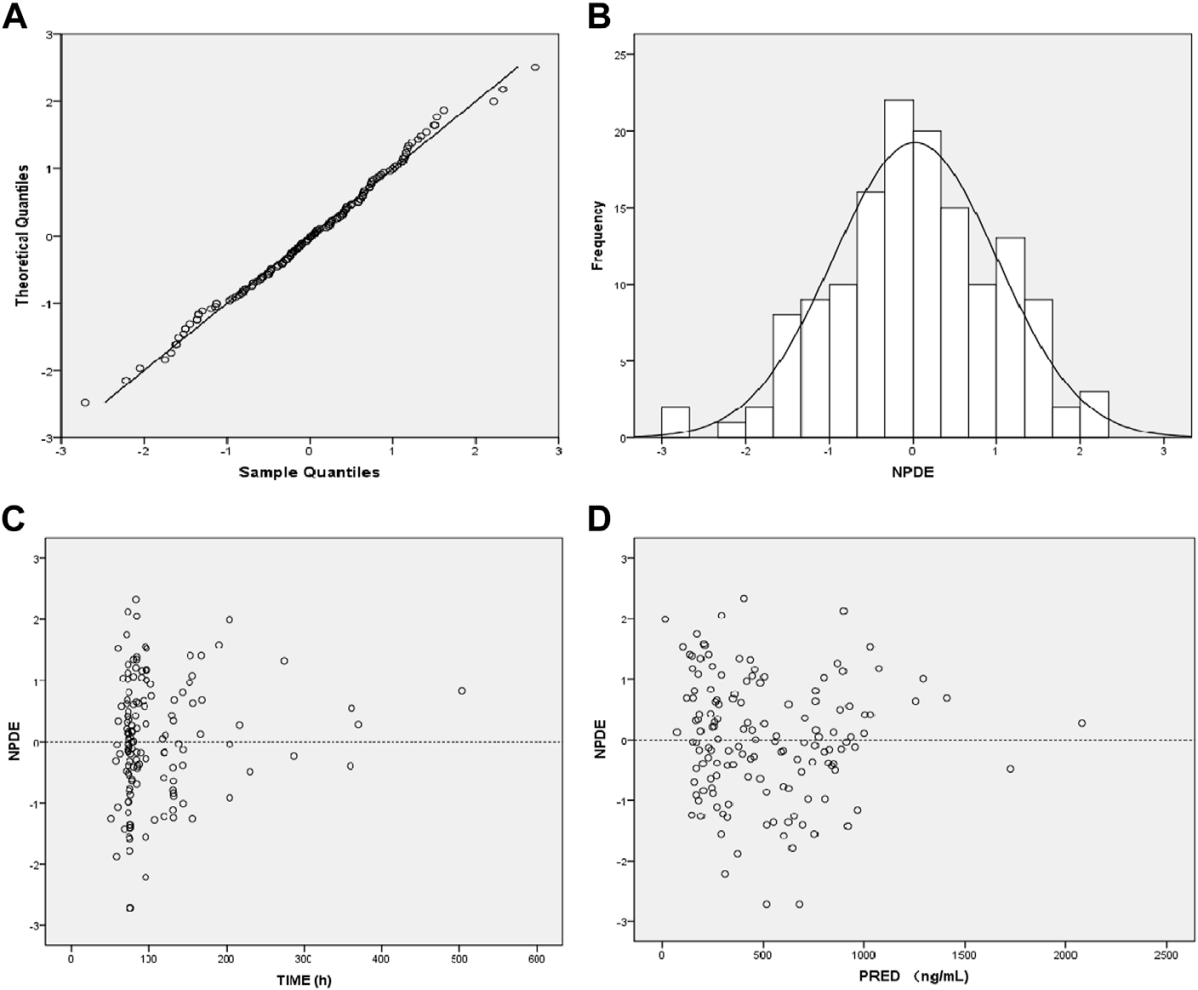
Diagnostic plot of the normalized prediction distribution error (NPDE) of the final model. **(A)** Q-Q plot of NPDE, **(B)** distribution histogram of NPDE, **(C)** Scatter plot of NPDE-TIME, **(D)** Scatter plot of NPDE-Population Predicted Values.

The GOF indicated that the observed concentration vs. PRED and IPRE data were evenly distributed on both sides of the Y = *X*-axis in the final model, with good consistency. The CWRES distribution was symmetric and most of the values fell within −2 and +2, and there were no values outside of −4 and +4 (Figure 1). The model was verified using the bootstrap method. This was performed 500 times, and 442 iterations were successful (88.4% robustness rate). The estimated parameters of the final model were close to the median values obtained through bootstrapping, the relative deviation was small and all values fell within the 95% CI. The established model was relatively stable (Table 2). In the final model, only the APACHEII score was supported as a linear covariate of tigecycline CL. After APACHEII score was added, the predicted and corrected VPC and NPDE had good GOF and excellent prediction performance (Figures 2, 3).

**TABLE 2:** PK parameters and bootstrap results of final model.

Parameter	Value	Bootstrap		Relative bias(%)
		Median	95%CI	
Theta1	11.30	10.90	8.75–13.40	3.54
Theta2	105.00	100.30	54.7–160.0	4.47
Theta3	0.14	0.13	0.03–0.21	7.14
Theta4	0.0059	0.0049	0.0033–0.0079	16.94
Omega1	0.065	0.060	0.021–0.106	7.69
Omega2	0.160	0.153	0.018–0.0483	4.37
Sigma1	0.0316	0.0314	0.0063–0.048	0.63

CI, confidence interval; Theta1, population typical value of clearance; Theta2, Population typical value of distribution volume; Theta3, population typical value of APACHE II-CL; Theta4, population typical value of AGE-V; Omega1, interindividual variability of clearance; Omega2, interindividual variability of distribution volume; Sigma1, residual variability.

### 3.3 Dose simulation

#### 3.3.1 MIC distribution

The distribution of strains are listed in Table 3, including 34,389 strains of *Acinetobacter* baumannii, 79,232 of *Klebsiella pneumoniae* and 108,666 of *Escherichia coli*.

**TABLE 3:** MIC distribution of tigecycline about three pathogens.

MIC (mg/L)	*Acinetobacter baumannii*		*Klebsiella pneumoniae*		*Escherichia coli*	
	Number of strains	Percentage (%)	Number of strains	Percentage (%)	Number of strains	Percentage (%)
0.06	2,153	6.26	300	0.38	17,910	16.48
0.125	5,370	15.62	3,130	3.95	46,735	43.01
0.25	6,025	17.52	21,790	27.50	30,621	28.18
0.5	7,078	20.58	31,110	39.26	9,804	9.02
1	7,622	22.16	13,484	17.02	2,725	2.51
2	4,106	11.94	6,081	7.67	825	0.76
4	1,683	4.89	2,872	3.62	40	0.04
8	352	1.02	465	0.59	6	0.01
total	34,389	100	79,232	100	108,666	100

MIC, minimal inhibitory concentration.

#### 3.3.2 PTA of tigecyclinee in different dosing regimens

**FIGURE 4:**
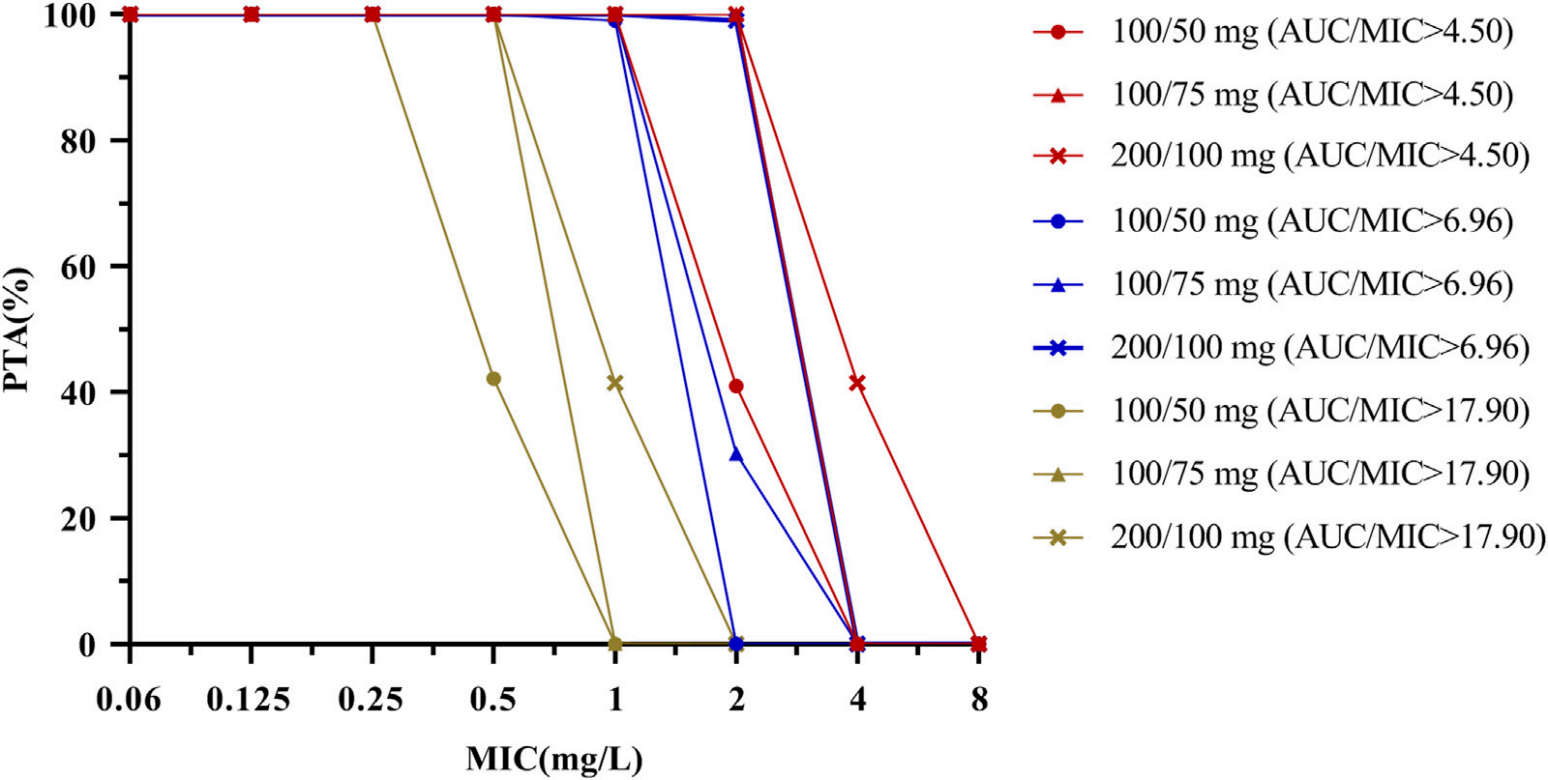
PTA by MIC for simulations of different dosing regimens of tigecycline for a PK/PD target of AUC_0–24_/MIC ≥ 4.5, 6.96 and 17.9. The PK/PD target is achieved when the probability of target attainment (PTA) is 90% coverage. PK parameter uncertainty (see Table 2) should be considered when using this figure.

PTA *versus* MIC profiles that corresponded to Monte Carlo simulations of different dose regimens for three PK/PD targets (AUC_0–24_/MIC ≥4.5, ≥6.96, and ≥17.9) are represented in Table 4; Figure 4. The results indicated that considering a target AUC_0–24_/MIC of ≥4.5, more than 90% of the patients with HAP would be successfully treated for bacteria in all three dose regimens with MIC ≤1 mg/L. The PTA value of the standard dose regimen (100 mg loading dose followed by a 50 mg maintenance dose at q12 h) was 40.96% with an MIC of 2 mg/L in patients with HAP; the ideal effect can be achieved by increasing the dosage. Considering a target AUC_0–24_/MIC of ≥6.96, the three dose regimens could be used to treat bacteria with MIC ≤1 mg/L in patients with cIAI. Regarding an MIC of 2 mg/L, only a higher maintenance dose of 100 mg at q12 h reached the ideal efficacy, and the other two regimens were not applicable. Finally, a target AUC_0–24_/MIC of ≥17.9 reached 100% in patients with cSSSI in the three tigecycline dose regimens, considering MIC ≤0.25 mg/L.

**TABLE 4:** PTA values of three tigecycline dosing regimens at different MIC.

Infection types	Dosage regimen	AUC_0-24_ (mg·h/L)	PK/PD target (AUC_0-24_/MIC)	PTA(%)							
				0.06	0.125	0.25	0.5	1	2	4	8
HAP	100/50 mg, q12 h	8.85	>4.50	100	100	100	100	100	40.96	0	0
	100/75 mg, q12 h	13.27	>4.50	100	100	100	100	100	100	0.07	0
	200/100 mg, q12 h	17.70	>4.50	100	100	100	100	100	100	41.48	0
cIAI	100/50 mg, q12 h	8.85	>6.96	100	100	100	100	99.07	0	0	0
	100/75 mg, q12 h	13.27	>6.96	100	100	100	100	100	30.29	0	0
	200/100 mg, q12 h	17.70	>6.96	100	100	100	100	100	99.02	0	0
cSSSI	100/50 mg, q12 h	8.85	>17.90	100	100	100	42.18	0	0	0	0
	100/75 mg, q12 h	13.27	>17.90	100	100	100	100	0.1	0	0	0
	200/100 mg, q12 h	17.70	>17.90	100	100	100	100	41.48	0	0	0

AUC_0-24_/MIC, ratio of the 24-h area under the curve to the MIC; PK/PD, Pharmacokinetics/pharmacodynamics; PTA, probability of target attainment; HAP, hospital acquired pneumonia; cIAI, complicated intra-abdominal infections; cSSSI, complicated skin and skin-structure infections.

#### 3.3.3 CFR of tigecyclinee for different pathogens under 3 dosing regimens

**FIGURE 5:**
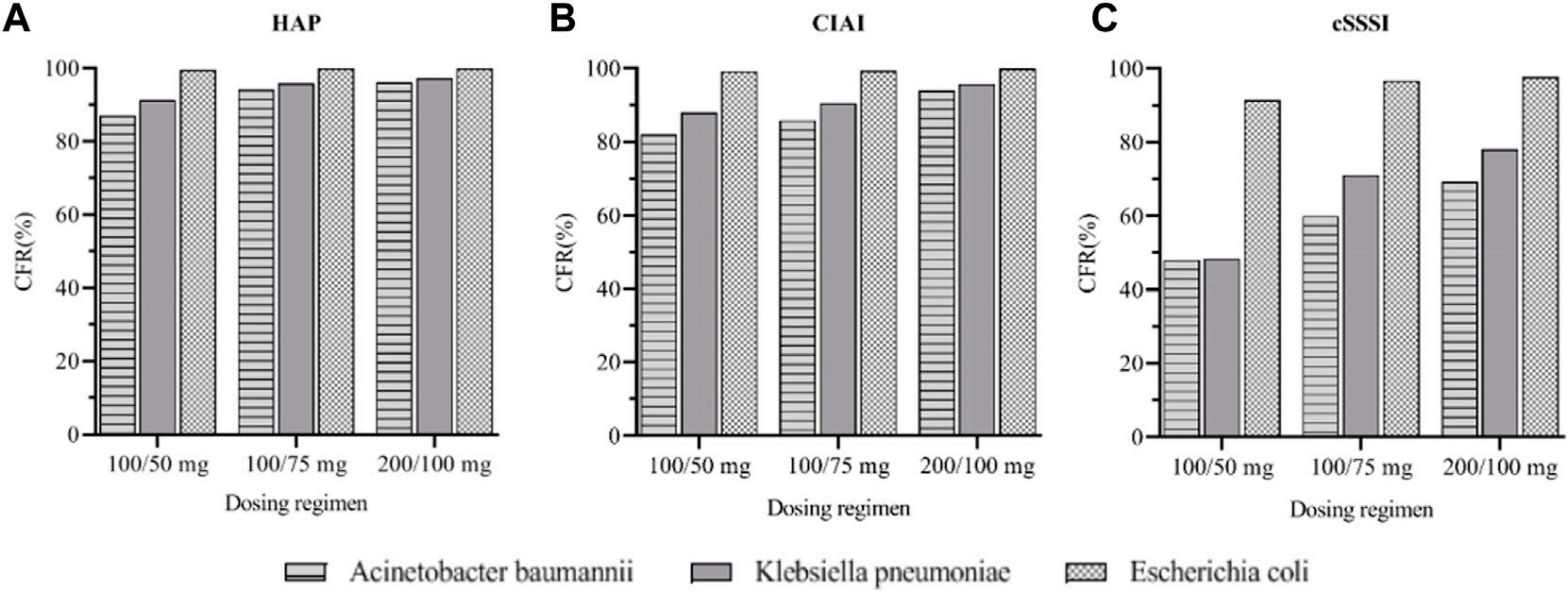
Cumulative fraction of response (CFR) of three tigecycline dosages regimens in different sites of infection. **(A)**: CFR for three Gram-negative bacteria under different dosages regimens of HAP **(B)**: CFR for three Gram-negative bacteria under different dosages regimens of cIAI **(C)**: CFR for three Gram-negative bacteria under different dosages regimens of cSSSI.

Monte Carlo simulation results indicated that for HAP, cIAI, and cSSSI infections caused by *A. baumannii*, the efficacy was poor when a standard dosage was used. When the maintenance dose was 100 mg, the CFRs of patients with HAP and cIAI were 96.11% and 93.97%, respectively, suggesting that increased doses should be considered in this group. No dose adjustment was needed for *K. pneumoniae* for AUC_0–24_/MIC targets of 4.5 and 6.96, and the three dose regimens almost all reached 90%. However, the efficacy of the three dose regimens was poor for patients with cSSSI, and it is necessary to consider the combination of drugs or different drugs to improve the effective rate. For the infections caused by *E. coli*, the CFR of the three dose regimens were all higher than 90%, indicating that tigecycline is more sensitive to infections caused by *E. coli* (Figure 5).

## 4 Discussion

Clinical efficacy and safety are the gold standard in antibiotic evaluations, and PK and PD play increasingly important roles in antibiotic selection. Understanding PK and PD is necessary for the formulation of drug delivery schemes, which can optimize tigecycline treatment to the maximum extent and reduce antibiotic resistance.

Most patients had pulmonary infections and multiple associated syndromes in this study. Considering that the PK characteristics of tigecycline may be affected by different disease states, various possible covariates (e.g., sex, age, weight, department distribution, liver function index, kidney function index, APACHEII score, course of treatment, combination of hepatotoxic drugs, and combination of antifungal treatment) were considered for selection. The preliminary screening results indicate that many possible covariates proposed in advance had no significant impact on the fixed-effect parameters in this study. In this study, the final PPK model indicates that APACHEII score and age will affect the CL and apparent Vd, respectively, of tigecycline in patients with severe illness. It was speculated that APACHEII score may be related to the special pathophysiological conditions of patients, while age to the drug distribution in patients, and liver and kidney functions. Our study was consistent with that of Xie et al. (2017), and there was no correlation between patient weight and tigecycline CL. Previous studies found that age, BMI, AST, and CLcr affect tigecycline CL and weight affects Vd in ICU patients (Borsuk-De Moor et al., 2018; Broeker et al., 2018; Zhou et al., 2021). However, the influence of weight on PK parameters was not observed in this study, which may be related to the small sample size of our study population and the age distribution of the enrolled population (the median age was 72 years, and the overall distribution was relatively concentrated). Because CL can be easily transformed into AUC, our model combined with the PK/PD target of tigecycline can provide accurate individualized treatment plans for clinical practice.

Tigecycline is a time-dependent antibacterial agent with a long post-antibiotic effect, and its PK/PD parameter is AUC/MIC. Some scholars believe that increased bacterial MIC and insufficient clinical tigecycline dosages are the main reasons for its poor clinical treatment effects. Our study therefore also evaluated the compliance of three PK/PD targets for different dose regimens. The results indicated that the PTA and CFR increased as the tigecycline dose increased and the MIC decreased. For patients with cSSSI caused by *A. baumannii* and *K. pneumoniae*, the CFRs of all dose regimens were less than 90%, so it is recommended to use a different drug or to use multiple drugs in combination. A meta-analysis found that for patients with severe blood flow infection, tigecycline combination therapy had lower mortality and more advantages than single drug therapy (Wang et al., 2017). For patients with HAP or cIAI caused by *A. baumannii* or *K. pneumoniae*, the tigecycline dosage needs to be increased to achieve the ideal therapeutic effect. Our study found that when treating multidrug-resistant bacterial infections, high-dose tigecycline had higher clinical efficiency, lower mortality and lower safety, which was consistent with the findings of Falagas et al. (2014). The clinical increase in tigecycline dosage is mostly limited by adverse drug reactions. Some studies have found that the gastrointestinal adverse reactions of tigecycline are related to eating. The single dose that patients can tolerate after eating can be increased from 100 mg to 200 mg (Kasbekar, 2006). It is worth noting that the PTA of tigecycline in the routine recommended dose regimen is less than 90% with an MIC of 2 mg/L, which may be related to the PK distribution characteristics of tigecycline in different parts of the human body and the physiological differences of the population.

The final model indicated that APACHEII score could affect the CL of tigecycline, which was still the advantage of this study. However, there were also some limitations: 1). the small number of patients enrolled and the large number of covariates included in the study led to more-stringent exclusion criteria, and so our final model may not fully represent all conditions of ICU patients, and 2). this study had a retrospective design and the sampling points of plasma concentration measurements were relatively sparse, which may not necessarily correspond to the concentration at the target site. We therefore intend to conduct a prospective study with intensive sampling to address the limitations in the current study.

## 5 Conclusion

In the PPK model of tigecycline established in this study, APACHEII score and age affected the CL and Vd of tigecycline, respectively. The evaluation and validation indicated a good fit to the data and an excellent prediction performance. The standard dose regimen of tigecycline (100 mg loading dose followed by a 50 mg maintenance dose at q12 h) was often not able to obtain satisfactory therapeutic effects for critically ill patients. For patients with HAP and cIAI caused by one of three pathogens, the efficacy rate can be improved by increasing the dose. This study provides a basis for the adjustment of the therapeutic dose of tigecycline for patients with severe nosocomial infection to ensure the antibacterial effect of tigecycline and reduce potential adverse drug reactions and drug resistance in the future.

## Data Availability

The raw data supporting the conclusion of this article will be made available by the authors, without undue reservation.
