# Supplementary material for: Population pharmacokinetics of tigecycline in critically ill patients
Source: Front Pharmacol. 2023 Mar 13;14:1083464. doi: 10.3389/fphar.2023.1083464 (PMC10040605; doi:10.3389/fphar.2023.1083464)
Supplement: Supplementary file 1 [file DataSheet1.PDF]

Appendix 1：

1.1 Investigation of a methodology for the determination of tigecycline in plasma by LC-MS/MS

The LC-MS/MS method established in this study was verified by specificity, linear range, precision, accuracy, recovery and stability, and proved to be stable and reliable. The linear relationship of tigacycline was good in the range of 1~2000 ng/mL, the lowest limit of quantitation was 1 ng/mL, and the regression equation was y = 0.0085x-0.1913. Correlation coefficient R^2 = 0.9907. Intra-day precision, inter-day precision and recovery of tigecycline at low concentration (100 ng/mL), medium concentration (500 ng/mL) and high concentration (1000 ng/mL) were analyzed, respectively. The Relative Standard Deviation (RSD) of inter-day precision under low, medium and high concentrations were 2.74%, 2.01% and 2.94%, respectively. RSD of intra-day precision were 4.15%, 2.64% and 3.23%, respectively. The extraction recoveries were 97.36%, 98.74% and 104.66%, respectively. The results are shown in Table 1-1 and Table 1-2.

The specificity of the method was verified by analyzing for the presence of interfering substances. Standard plasma samples (tigecycline: range of concentrations; Internal standard: 100 ng/mL) are shown in Figure 1-1, and plasma samples of subjects are shown in Figure 1-2. The retention time of tigacycline and internal standard was not observed to be the same as that of the endogenous substance, which could prove that the endogenous substance in plasma had no effect on the assay results.

Table 1-1. Precision results of the determination of tigecycline in patients' plasma by LC-MS/MS

	Concentration (ng/mL)	RSD (%)
Inter-day precision	100	2.74
	500	2.01
	1000	2.94
Intra-day precision	100	4.15
	500	2.64
	1000	3.23

RSD, Relative Standard Deviation.

Table 1-2. Results of extraction recovery rate of tigecycline plasma sample

Concentration (ng/mL)	Rate of recovery (%)
100	97.36%

500	98.74%
1000	104.66%

RSD, Relative Standard Deviation.

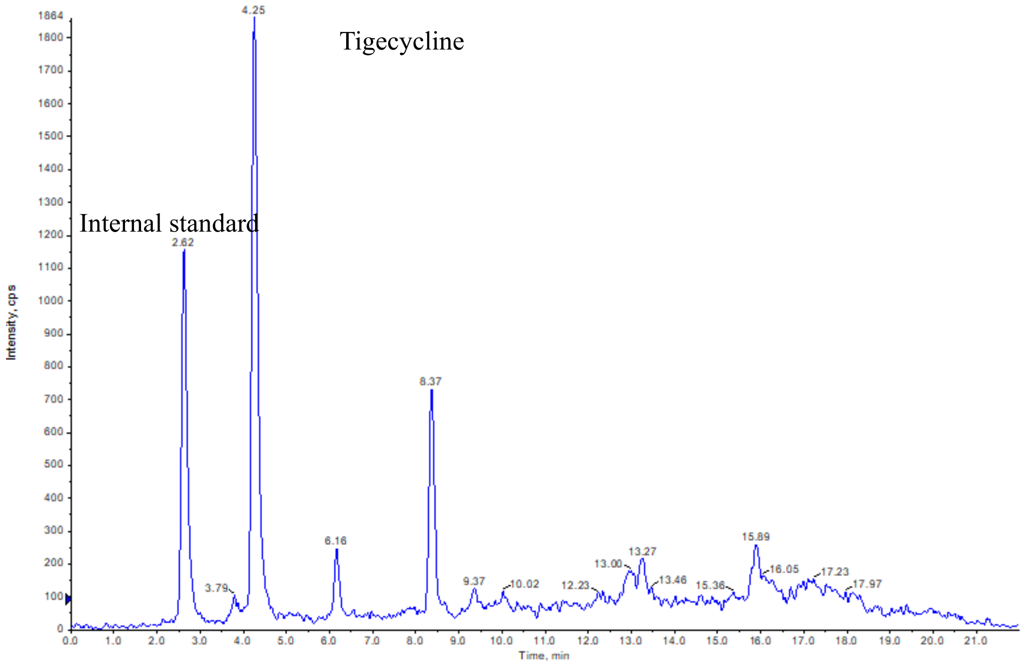

Figure 1-1. Chromatogram of blank plasma added with tigecycline and internal standard (100 ng/mL)

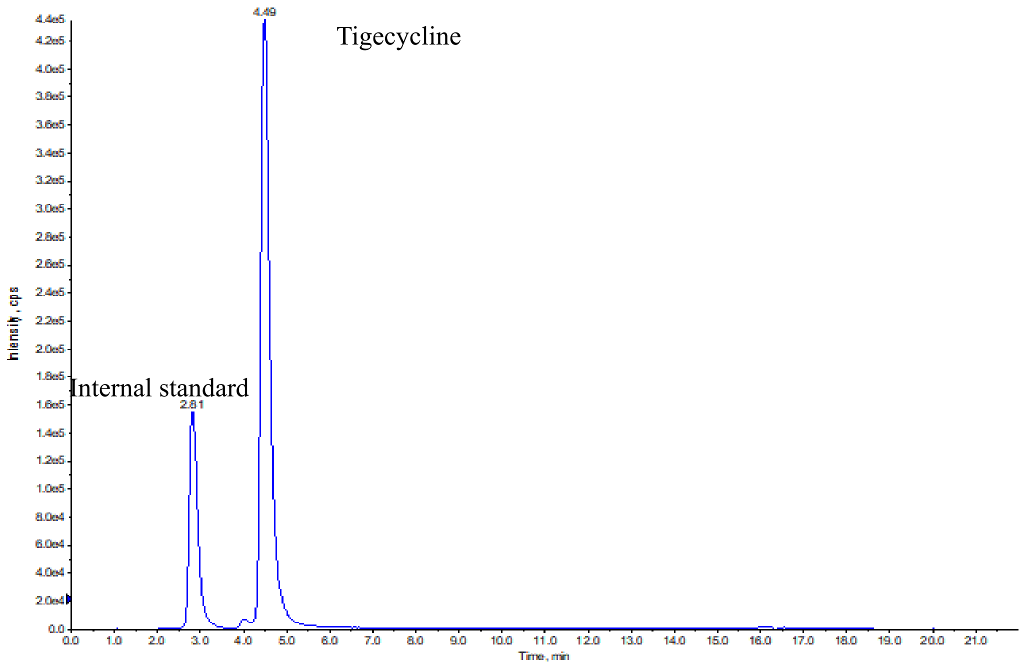

Figure 1-2. Chromatogram of plasma sample after stable state of tigecycline after intravenous infusion of 100 mg for the first time and then 50 mg for 12 hours

Appendix 2：

2.1 Investigation of covariable model

The blood concentration of tigecycline in ICU patients was taken as PK index, and the age, sex, body weight, liver function index (ALT, AST, ALP, TB), renal function index (CLCR, BUN), ALB, APACHE II score, whether anti-fungal treatment, and sodium ion were taken as covariates to investigate the relationship between these covariates and fixed effects (CL, V) and random effects (ETA1, ETA2), as shown in Figure 2-1.

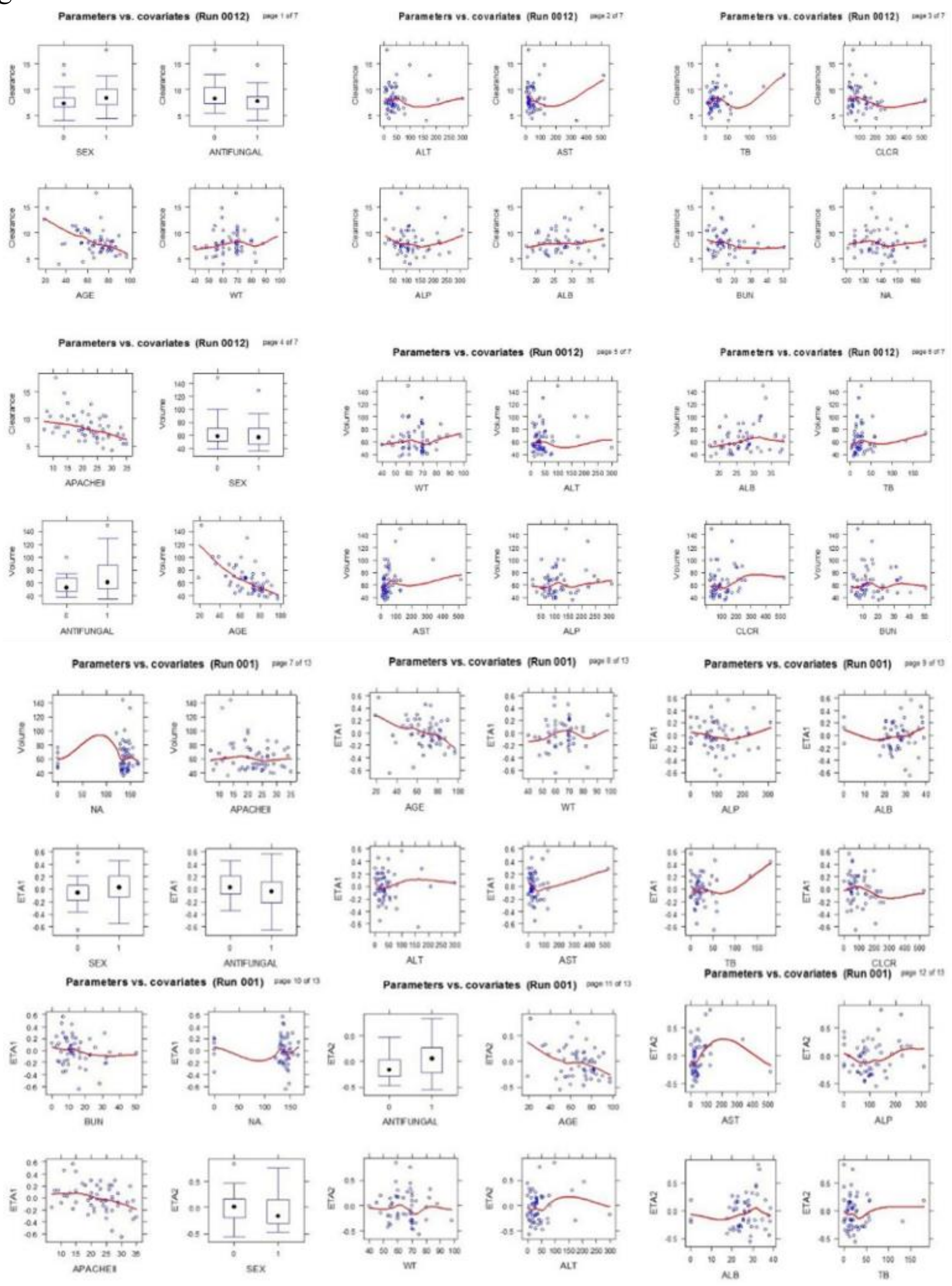

Figure 2-1. Relationship between covariates and fixed effect parameters and random effect parameters

2.2Total regression model

The total regression model is to add covariates one by one on the basis of the basic model. In the first round of forward inclusion, the delta OFV decreased by more than 3.84 in the model 2-9, in which APACHE II as the covariate of CL, the OFV value decreased the most. Then, in the second round of inclusion, the covariates are gradually added on the basis of model 3, and the results of model 10 and 11 are statistically significant. In the third round of forward inclusion, the OFV value of model 12 dropped the most, and the final total regression models were models 3, 5, and 7 (Table 2-1). See Table 2-1 for details.

Table 2-1. Forward inclusion of covariate process

NM-model	Parameter- Covariate	OFV	Delta OFV	*P* value	Sig(Yes or No)
1	Base-model	1540.83	-	-	-
2	AGE-CL	1536.68	4.15	<0.05	Yes
3	APACHE II-CL	1531.22	9.61	<0.01	Yes
4	ALB-CL	1536.63	4.2	<0.05	Yes
5	TB-CL	1535.45	5.38	<0.05	Yes
6	BUN-CL	1536.28	4.55	<0.05	Yes
7	AGE-V	1532.35	8.48	<0.01	Yes
8	ALP-V	1535.77	5.06	<0.05	Yes
9	CLCR-V	1536.16	4.67	<0.05	Yes
10	APACHE II-CL＆ TB-CL	1527.14	13.69	<0.001	Yes
11	APACHE II-CL＆ AGE-V	1523.93	16.9	<0.001	Yes
12	APACHE II-CL＆ TB-CL＆AGE-V	1519.44	21.39	<0.001	Yes
Total regression model: APACHE II-CL＆TB-CL＆AGE-V					

OFV, Objective function value; Sig, Significant difference; Yes, Preserve covariates; No, Remove covariates.

2.3The establishment of the final model

On the basis of the complete total regression model, the covariates are tested one by one by using the reverse elimination method. If the change of OFV value after removing a covariate is greater than 6.64 ($P < 0.01$, $df = 1$), the covariate will be retained, and vice versa. See Table 2-2 for the final model parameters.

Table 2-2. Reverse elimination method to establish final model

NM-model	Parameter-Covariate	OFV	Delta OFV	*P* value	Sig(Yes or No)
12	APACHE II-CL＆	1519.44	-	-	-

	TB-CL＆AGE-V				
13	APACHE II-CL＆ TB-CL	1527.14	7.70	<0.01	Yes
14	APACHE II-CL＆ AGE-V	1523.93	5.51	>0.01	No
15	TB-CL＆AGE-V	1529.36	9.92	<0.01	Yes
Total regression model: APACHE II-CL＆AGE-V					

OFV, Objective function value; Sig, Significant difference; Yes, Preserve covariates; No, Remove covariates.
